# An Assessment of Mentions of Adverse Drug Events on Social Media With Natural Language Processing: Model Development and Analysis

**DOI:** 10.2196/38140

**Published:** 2022-09-28

**Authors:** Deahan Yu, V G Vinod Vydiswaran

**Affiliations:** 1 School of Information University of Michigan Ann Arbor, MI United States; 2 Department of Learning Health Sciences Medical School University of Michigan Ann Arbor, MI United States

**Keywords:** natural language processing, machine learning, adverse drug event, pharmacovigilance, social media, drug, clinical, public health, health monitoring, surveillance, drug effects, drug safety

## Abstract

**Background:**

Adverse reactions to drugs attract significant concern in both clinical practice and public health monitoring. Multiple measures have been put into place to increase postmarketing surveillance of the adverse effects of drugs and to improve drug safety. These measures include implementing spontaneous reporting systems and developing automated natural language processing systems based on data from electronic health records and social media to collect evidence of adverse drug events that can be further investigated as possible adverse reactions.

**Objective:**

While using social media for collecting evidence of adverse drug events has potential, it is not clear whether social media are a reliable source for this information. Our work aims to (1) develop natural language processing approaches to identify adverse drug events on social media and (2) assess the reliability of social media data to identify adverse drug events.

**Methods:**

We propose a collocated long short-term memory network model with attentive pooling and aggregated, contextual representation generated by a pretrained model. We applied this model on large-scale Twitter data to identify adverse drug event–related tweets. We conducted a qualitative content analysis of these tweets to validate the reliability of social media data as a means to collect such information.

**Results:**

The model outperformed a variant without contextual representation during both the validation and evaluation phases. Through the content analysis of adverse drug event tweets, we observed that adverse drug event–related discussions had 7 themes. Mental health–related, sleep-related, and pain-related adverse drug event discussions were most frequent. We also contrast known adverse drug reactions to those mentioned in tweets.

**Conclusions:**

We observed a distinct improvement in the model when it used contextual information. However, our results reveal weak generalizability of the current systems to unseen data. Additional research is needed to fully utilize social media data and improve the robustness and reliability of natural language processing systems. The content analysis, on the other hand, showed that Twitter covered a sufficiently wide range of adverse drug events, as well as known adverse reactions, for the drugs mentioned in tweets. Our work demonstrates that social media can be a reliable data source for collecting adverse drug event mentions.

## Introduction

### Background

Adverse reactions to drugs are among the most significant concerns in both clinical practice and public health monitoring, but they do not have a consistent definition in the literature. According to Edwards and Aronson [1], side effects of a particular drug are defined as “unintended effects related to the pharmacological properties occurring at normal dose” of the drug. Unintended effects can be either harmful or beneficial. For example, β-blockers are mainly used for hypertension, but they can also relieve chest pain (or angina) in patients [1]. According to a World Health Organization (WHO) report [2], adverse reactions are defined as “any response to a drug that is noxious, is unintended, and occurs at doses normally used in humans.” A similar definition of adverse reaction was used by Asscher et al [3] and Pirmohamed et al [4], except that their definitions included the condition that the drug was used in its proper clinical application. In other words, the WHO definition allows for an improper use of a drug with a normal dose, while Asscher et al and Pirmohamed et al do not include such cases. A definition of adverse reactions by Karch and Lasagna [5] is similar but includes the effects of intentional overdoses and drug abuse. Although various definitions of adverse reactions are used, the most common component of these definitions is unintended consequences caused by or suspected to be due to the use of a drug [1-5].

Adverse events, on the other hand, are defined as “untoward occurrences following exposure to a drug but not necessarily caused by the drug” [1,3]. While the terms “adverse event” and “adverse reaction” are similar, they cannot be used interchangeably, because there is no causality assumption in the definition of adverse events, while there is a causality assumption in the definition of adverse reactions. Adverse reactions are reported to be among the top 10 leading causes of death [6,7]. To increase postmarketing surveillance of drugs and improve drug safety, multiple measures have been put into place. These include implementing spontaneous reporting systems, such as the US Food and Drug Administration Adverse Events Reporting System (FAERS) [2,7,8].

On the other hand, researchers have also looked at developing automated systems that use electronic health records and social media data [9-11] to collect experiences of adverse events that can be further investigated as possible adverse reactions. Recently, deep neural network–based models have been developed to detect adverse events in tweets [12-14]. Long short-term memory (LSTM) networks and pretrained language models, such as bidirectional encoder representations from transformers (BERT) [15] and generative pretraining language models [16], have been chosen as models for this application [12-14]. However, there is still room for improvement in the implementation of such systems [9-11]. Various neural network systems have been presented by other researchers, but no system to date incorporates both recurrent-based networks (eg, LSTM) and attention-based networks (eg, BERT). Capturing both sequentially processed output and contextually processed output could help the model better learn the data and the task. Lastly, machine learning and deep learning models have shown their effectiveness at detecting adverse event mentions in social media data [17], but it is still uncertain whether social media are valid as a data source for the purpose of adverse event detection.

### Goal of This Study

In this paper, we use the term “adverse drug event” (ADE) rather than “adverse event.” We formulated the task of identifying ADE mentions from tweets as a classification task, that is, labeling tweets based on whether or not they contain a mention of an ADE. We propose a neural network–based framework that incorporates augmented medical representation and contextual representation to build a robust classification model. Our work aims to develop a natural language processing (NLP) system that identifies ADE mentions based on social media texts and to assess the reliability of social media data, especially Twitter, as a means to collect that information. Our research questions are as follows: “Could contextual representation from a pretrained language model help enhance a model for classifying ADE tweets?” and “Could social media be a reliable data source to collect mentions of ADEs?”

We conducted a comprehensive experimental analysis to validate the effectiveness of the model. In addition, we performed a systematic evaluation study to determine the reliability of Twitter as a data source for collecting mentions of ADEs. Our work makes the following empirical contributions: (1) we demonstrate that incorporating contextual representations with augmented medical representations significantly improves the performance of the adverse event detection task compared to not incorporating contextual representations, (2) we show that the current automated systems to identify mentions of ADEs in tweets are not sufficiently generalizable, and (3) we observe that Twitter covers a sufficiently wide range of ADEs relatively well, including known ADEs, and conclude that social media can be a reliable data source for collecting ADE mentions.

### Related Work

Before a drug is released to market, an initial description of related ADEs is obtained through randomized controlled trials [18]. These trials may provide an initial description that is not fully complete [19]. Due to the incompleteness of the initial list of ADEs, pharmacovigilance plays a significant role in the postmarketing phase and is necessary to collect any new information on ADEs. Social media, including Twitter, have been explored as platforms for pharmacovigilance, such as by collecting mentions of ADEs through NLP [11,17,20-22]. The text of tweets is relatively short but still conveys information about patient experiences that are often self-disclosed. For a tweet to be considered ADE-related, the tweet must not only mention at least one adverse event, but must also mention a drug by name. Notably, a tweet cannot be considered ADE-related if there is no mention of drugs.

Data sets of labeled tweets for identifying mentions of ADEs have been developed to benchmark NLP systems in shared competitions [11-13,22-25]. These annotated data sets have allowed researchers to develop automated systems and compare them against each other. Early systems for identifying mentions of ADEs in tweets were based on curated lexicons, heuristic rules, pattern matching, or supervised machine learning approaches [23,24]. Various dictionary-based features, such as ADE lexicons, drug names, and medical concepts were explored, along with linguistic and sentiment analysis. Recently, neural network–based models have been a popular choice due to their outstanding performance [11].

## Methods

### Data Sources

We used 3 Twitter-based data sets to develop and evaluate our models—1 for training and 2 for evaluation. The training set and the first evaluation set were obtained from a shared task for automatic classification of English-language tweets that report adverse effects, organized as part of the 2020 Social Media Mining for Health (SMM4H) workshop [11]. According to the organizers of the shared task, the tweets were collected via Twitter’s public streaming API. Generic and trade names for drugs, along with their common misspellings, were used as keywords to collect data. After the collection, the tweets were annotated independently by 2 annotators, with a Cohen κ for interannotator agreement of 0.82. Tweets with disagreements were reannotated until the pair reached consensus. The second evaluation set was obtained from a publicly available reference data set called WEB-RADR (web-recognizing adverse drug reactions), developed by Dietrich et al [22]. These tweets were collected in a similar fashion. Annotations were done by 2 teams of 9 annotators each; however, no measures for interannotator agreement are reported.

Table 1 summarizes the statistics of the 3 data sets. After preprocessing and removing duplicate tweets, there were 24,700 tweets in the training set. Of these, approximately 9% (2362) of them were labeled as ADE tweets, that is, tweets containing 1 or more mentions of adverse events along with at least 1 mention of a drug. The remaining 91% (22,338) of tweets were labeled as non-ADE tweets, meaning that these tweets did not contain any mention of an adverse reaction but contained a drug mention. Of 24,700 tweets, 20,098 (81.4%) were used to train the models while the other 4602 (18.6%) were used for validation. The distribution of ADE versus non-ADE tweets was more skewed in the SMM4H evaluation set. Of the 4759 tweets in the evaluation set, only 194 (4.1%) were labeled as ADE tweets and 4565 (95.9%) were labeled as non-ADE tweets.

We also evaluated our models on WEB-RADR [22], which we used as a second, independent data set. The original data set consists of 57,473 tweets, with 1056 tweets (1.8%) labeled as ADE tweets and 56,417 (98.2%) as non-ADE tweets. However, from the original data set, we were able to successfully collect only 34,369 (59.8%) tweets, possibly due to suspended accounts or deleted tweets. Of these, 645 (1.9%) were labeled as ADE tweets, while the remaining 33,724 (98.1%) were non-ADE tweets.

All tweets were preprocessed to separate punctuation marks, remove special characters and URLs, replace user mentions beginning with @, and replace text emoticons with a normalized token. No specific text cleaning packages were used.

**Table 1:** Statistics for the training and evaluation data sets.

Data set	Tweets, N	ADE^a^ tweets, n	Non-ADE tweets, n	Unique drugs, n	Drugs in tweets but not in library, n
SMM4H^b^ training	24,700	2362	22,338	1020	31
SMM4H evaluation	4759	194	4565	688	129
WEB-RADR^c^ evaluation	34,369	645	33,724	685	25,646

^a^ADE: adverse drug event.

^b^SMM4H: Social Media Mining for Health.

^c^WEB-RADR: web-recognizing adverse drug reactions.

### NLP System Development

#### Model Selection

In recent years, pretrained language models have been widely deployed as base models for numerous NLP tasks that can be fine-tuned to a data set for a particular downstream task, often referred to as transfer learning. Despite relatively simple training, such transfer learning approaches have been shown to be powerful tools for many NLP tasks, including ADE classification. Transfer learning makes downstream tasks successful because these language models are trained on a large corpus; hence, they gain strong representational power.

**Figure 1:**
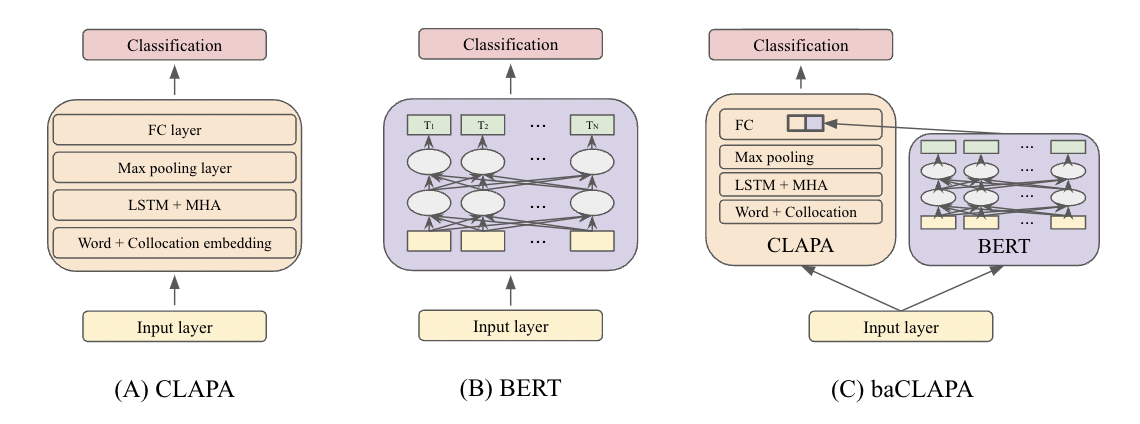
Schematic diagram of the 3 models that highlights how each model is configured. A: CLAPA; B: BERT; C: baCLAPA. baCLAPA: bidirectional encoder representations from transformers–assisted collocated long short-term memory with attentive pooling and aggregated representation; BERT: bidirectional encoder representations from transformers; CLAPA: collocated long short-term memory with attentive pooling and aggregated representation; FC: fully connected; LSTM: long short-term memory; MHA: multi-head attention.

In our previous work, we proposed a collocated LSTM model with attentive pooling and aggregated representation (CLAPA) that utilized neighborhood information to build a better representation of medical concepts [26]. The model focused on enhancing medical concepts by incorporating neighborhood information through a collocation graph. While CLAPA enriched the representation of medical concepts, it had relatively weak representation of other context information, such as semantics. The capability of a pretrained model to provide a robust representation of context information may help assist CLAPA to learn better. With this motivation, we extended CLAPA to BERT-augmented CLAPA (baCLAPA), which incorporated BERT’s logits with CLAPA’s trained representation. BERT was chosen because it was the most competitive model among pretrained models reported in the 2019 SMM4H task [13]. The 3 models compared in this task are illustrated in Figure 1 and summarized below.

#### CLAPA Model

CLAPA [26], illustrated in Figure 1A, uses collocation information to improve the representation of medical concepts. CLAPA requires three main components: (1) medical concepts, (2) a collocation graph, and (3) a model architecture.

First, for medical concepts, the generic names and brand names of medications were collected from MedlinePlus [27]. A few generic medical words or brand names, such as “Amen” and “Heather,” were removed to reduce noise. Then, the list of medical concepts was expanded by adding medical words from tweets in the training set that were missing in the drug list obtained from MedlinePlus. As a result, a total of 4888 medical concepts were collected, including 4747 drug names from MedlinePlus and 141 drug names from the SMM4H training set.

Second, for the collocation graph, each unique word in the training set was assigned as a node, and edges were added between node pairs if the corresponding pair of words were adjacent to each other. After the graph was constructed, the graph was reduced by retaining only the closest 15 neighbor nodes per medical concept, following an empirical analysis of neighborhood size [26].

Third, for the model architecture, LSTM networks with 4 layers and 300 input sizes were implemented, followed by 3 multi-head attention layers and max pooling and pooling layers. FastText pretrained embedding [28] was used for word embedding. All hyperparameters were jointly trained with a learning rate of 0.001 and a cross-entropy loss function.

#### BERT Model

As another baseline model, we instantiated a BERT model [15], illustrated in Figure 1B. The bert-base-uncased model was used for classification and was tuned based on the recommendation for hyperparameter settings [15]. The BERT model was fine-tuned on the training set without any further modification on hyperparameters. Two tokens, [CLS] and [SEP], were added to the beginning and end of the input representation. Each sentence was tokenized through BertTokenizer and fed into the BERT model. Our BERT model contained the same parameters as the base model, with 12 layers, 768 input sizes, and 12 multi-head attentions. The hyperparameters of the classification layer were jointly trained with a learning rate of 5*e*^–5^.

#### baCLAPA Model

Our proposed baCLAPA model is illustrated in Figure 1C. The model consists of 2 parallel stacks—a CLAPA model and a BERT model. The input sentence feeds into both the CLAPA and BERT models. Each network independently learns input embeddings. Once each model produces the final hidden states, the states are reduced to representations with a size of 2, which are commonly referred to as logits. The raw output representation of BERT is then incorporated into CLAPA, either as large as the final hidden states or as small as logits, mapped to a 2-dimensional vector space for a binary classification. In the task presented in this paper, BERT’s logits were used to assist CLAPA because logits provide a brief but comprehensive representation of how networks have learned from inputs. Thus, BERT’s logits were concatenated with CLAPA’s logits to generate predictions. In Figure 1C, 2 bold boxes inside the fully connected layer show how BERT’s logits and CLAPA’s logits are concatenated. Formally, this can be written as follows:




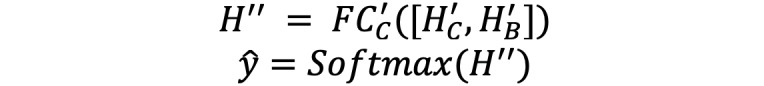




where 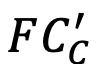 refers to the last fully-connected layer in CLAPA, 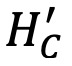 and 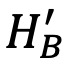 are logits from CLAPA and BERT, and 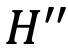 is the final logit. Once CLAPA and BERT produce their logits, BERT’s logits are passed to CLAPA. Then, a concatenation of their logits is fed into 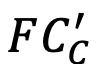, which produces 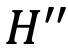. Finally, the final logits are fed into Softmax for binary classification.

### Baselines

Two additional models were used as baselines. First, we used an SVM model with a linear kernel, with other hyperparameters set to default values. The input representation included a term frequency–inverse document frequency weighted representation with trigram features. As a second baseline, we used a random model with weighted distribution.

### Validation Study to Determine the Reliability of Social Media as a Data Source

#### Study Questions

To validate the reliability of the social media data as a means to collect ADEs, we analyzed tweets that were collected by our baCLAPA model. This study aimed to answer two questions about social media data: (1) what kinds of ADEs are mentioned on Twitter? and (2) of the ADE mentions for each known drug on Twitter, how many also mentioned known adverse reactions listed in an authoritative source? Answering the first question would reveal how various kinds of ADEs are covered on social media, and answering the second would reveal how many relevant ADEs are mentioned on social media. The known adverse events were collected from MedlinePlus, an authoritative, popular, and credible website run by the US National Library of Medicine.

#### Obtaining ADE Tweets: Data Source and ADE Classification

The Twitter data used for this study were obtained from a paper by Vydiswaran et al [29]. The data were collected via the Twitter API, user timelines, and the Decahose stream, which is a 10% random sample of the real-time Twitter stream. First, the Twitter API and user timelines were used to collect all tweets from users near the Detroit metropolitan area. Then, the data set was expanded through the Decahose stream. In total, the data set contained 28.8 million tweets. More details about the data collection can be found in the paper by Vydiswaran et al [29].

First, the 28.8 million tweets were filtered through our drug list, which consisted of 4888 drug names. This step allowed us to sort out tweets containing at least one drug keyword. This let us identify 34,536 of 28.8 million tweets as drug-mentioning tweets. Then, our baCLAPA model was applied to those tweets and identified 1544 ADE tweets.

#### Qualitative Content Analysis of Tweets

We conducted a qualitative content analysis [30] to answer the two questions mentioned above: (1) how many different types of ADEs are covered on Twitter? and (2) how many ADE tweets about a particular drug identify an adverse event that is a known adverse reaction for that drug on MedlinePlus? We first extracted 139 unique drugs mentioned in the 1544 tweets. Then, we conducted a qualitative content analysis to derive themes for the ADEs within the 1544 tweets. During the qualitative coding process, we found that the drug word “caffeine” mostly referred to coffee and the word “vitamin” was too general to determine which vitamin supplement was taken. Therefore, tweets containing only these drug words were dropped—462 tweets for “caffeine” and 141 tweets for “vitamin”. A total of 941 ADE tweets were thus qualitatively analyzed. These tweets were manually coded to identify themes for ADEs until the themes were saturated. The themes were reviewed by a domain expert after the analysis was completed.

Once we identified the themes, we collected information about known adverse reactions for each drug through MedlinePlus and compared them against themes identified by the content analysis. For example, when analyzing ADE tweets about ibuprofen, we identified two themes: nausea and sweating. When reviewing information about ibuprofen on MedlinePlus, we only found relevant mentions of ibuprofen potentially causing nausea, and did not find any sweating-related adverse reactions. Thus, ibuprofen was paired with the nausea-related ADE theme as a known adverse reaction but not with the sweat-related ADE theme. This way, we linked all ADE tweets and known adverse reactions to a particular drug to each ADE theme.

## Results

### Experimental Results of the NLP System

We first present the performance of the models on the validation set. This allows us to compare the overall performance of the models, including the baselines. Both CLAPA and baCLAPA were evaluated on the SMM4H evaluation set [31]. We further evaluated the models on another data set, the WEB-RADR evaluation set, to validate whether the extended models performed better than the original models on various data sets.

As shown in Table 2, the random and SVM baseline models did not outperform the neural network–based models, but the recall score for the SVM model was the second highest. Of all models, baCLAPA performed the best for all performance metrics: precision, recall, and F1. On average, it performed about 0.026 F1 points better than CLAPA on the validation set.

To further evaluate our method, we picked the best CLAPA and baCLAPA models from the 10 validation runs. Their performance on the validation set is shown in the first 2 result rows of Table 3. On both evaluation data sets, baCLAPA outperformed CLAPA on the F1 metric. Precision and recall values are not available for CLAPA on the SMM4H evaluation set because it was used only for the best (baCLAPA) model [31]. While baCLAPA performed better for F1 score than CLAPA on the SMM4H evaluation set by 0.07, the improvement was relatively small on the WEB-RADR evaluation set. Most of this improvement was attributable to the significantly higher recall. CLAPA outperformed baCLAPA on the precision measure on WEB-RADR.

**Table 2:** Average performance of 10 runs on the validation set. Italics represent the best model for each performance metric.

Model	Precision (SD)	Recall (SD)	F1 score (SD)
Random	0.099 (0.01)	0.103 (0.01)	0.101 (0.01)
SVM^a^	0.386 (0)	0.638 (0)	0.481 (0)
CLAPA^b^	0.581 (0.03)	0.623 (0.03)	0.599 (0.01)
BERT^c^	0.54 (0.03)	0.602 (0.04)	0.567 (0.01)
baCLAPA^d^	*0.603* (0.02)	*0.652* (0.03)	*0.625* (0.007)

^a^SVM: support vector machine.

^b^CLAPA: collocated long short-term memory with attentive pooling and aggregated representation.

^c^BERT: bidirectional encoder representations from transformers.

^d^baCLAPA: bidirectional encoder representations from transformers–assisted collocated long short-term memory with attentive pooling and aggregated representation.

**Table 3:** Evaluation of collocated long short-term memory with attentive pooling and aggregated representation (CLAPA) and bidirectional encoder representations from transformers–assisted CLAPA (baCLAPA) on 2 evaluation sets. Italics represent the best model for each performance metric.

Data set and model		Precision		Recall	F1 score
**Validation**					
	CLAPA^a^		0.563	0.649	0.603
	baCLAPA^b^		0.589	0.676	*0.629*
**SMM4H^c^ evaluation**					
	CLAPA		—^d^	—	0.44
	baCLAPA		0.48	0.54	*0.51*
**WEB-RADR^e^ evaluation**					
	CLAPA		0.356	0.386	0.371
	baCLAPA		0.334	0.479	*0.394*

^a^CLAPA: collocated long short-term memory with attentive pooling and aggregated representation.

^b^baCLAPA: bidirectional encoder representations from transformers–assisted collocated long short-term memory with attentive pooling and aggregated representation.

^c^SMM4H: Social Media Mining for Health.

^d^Not available.

^e^WEB-RADR: web-recognizing adverse drug reactions.

### Qualitative Content Analysis of ADE Tweets

Table 4 summarizes the top 7 ADE themes, shows the frequency of tweets for each theme, and provides paraphrased examples. The major thematic areas include mental health–related ADEs and sleep-related ADEs. Tweeters also frequently shared their experience of pain-related ADEs. The remaining themes were discussed less frequently in our data set.

**Figure 2:**
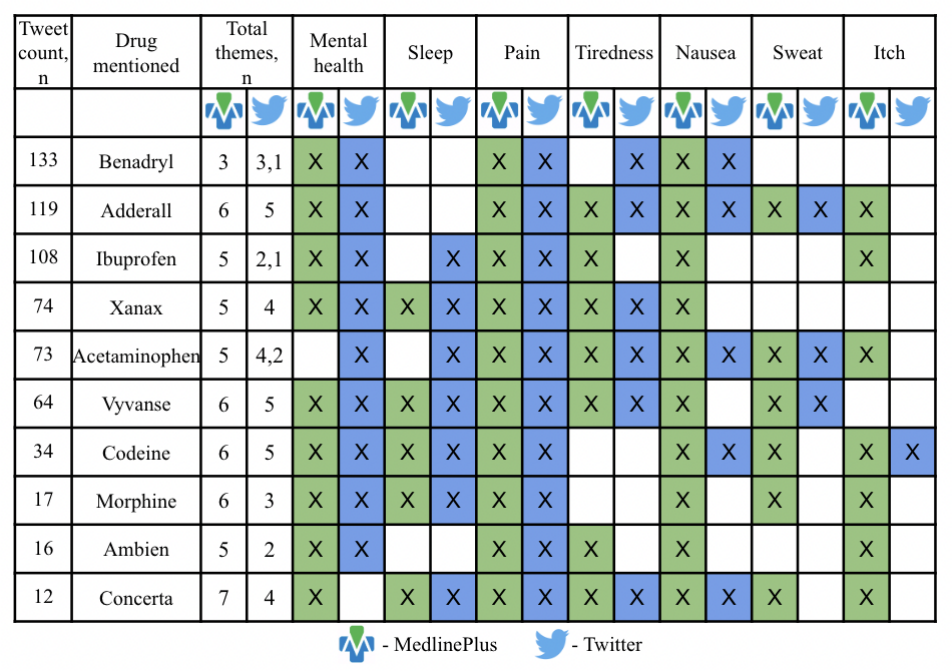
The top 10 drugs with known adverse reactions found in MedlinePlus versus adverse drug events found in tweets. X: drug with at least one known adverse reaction or adverse drug event related to a particular theme. Values before commas indicate themes mentioned in tweets as well as MedlinePlus, while values after commas indicate values indicated only in tweets.

Each row in Figure 2 represents a drug. In the 108 tweets for “ibuprofen,” mentions of 3 drugs are grouped together: Advil (n=40), ibuprofen (n=36), and Motrin (n=32); the 73 tweets for “acetaminophen” group together mentions of 2 drugs: Tylenol (n=71) and acetaminophen (n=2). The third column indicates the number of themes with known adverse reactions from MedlinePlus as well as the number of themes with ADE mentions on Twitter. The fourth column can have 2 different numbers, separated by a comma: the first is the number of themes that overlap with known adverse reactions, while the second, if present, is the number of themes that do not overlap with known adverse reactions. For example, Benadryl has 3 themes with known adverse reactions, all of which were captured in tweets, and 1 theme that was not listed in MedlinePlus but only mentioned in tweets. For Adderall, 6 themes contained known adverse reactions; 5 of these had related tweets.

**Table 4:** Top 7 adverse drug event themes with frequencies and examples (N=941).

Adverse drug event theme	Tweets, n (%)	Paraphrased examples
Mental health	204 (21.7)	Feeling emotionally unstable, depressed, or high
Sleep	201 (21.4)	Feeling sleepy, being knocked out by a drug, wanting to sleep, not being able to sleep, being able to stay awake at night
Pain	151 (16)	Experiencing other pains or aches, such as headache or stomachache
Tiredness	27 (2.9)	Feeling extremely tired
Nausea	21 (2.2)	Feeling nausea or a need to vomit
Sweating	20 (2.1)	Experiencing sweating
Itchiness	16 (1.7)	Feeling itchy

## Discussion

### Principal Results of the NLP System

By running our models on the validation set shown in Table 2, we confirmed that the performance of CLAPA was almost the same as that of previously published models, with an F1 score of 0.5998 [26]. The performance of BERT was also similar to that of BERT-based models reported in an overview of the SMM4H 2019 shared task [13]. This confirmation ensured that our results did not include any noise due to unexpected performance of the models. Our evaluation results demonstrate that baCLAPA outperformed CLAPA on both evaluation sets. However, we made two observations: (1) there was a significant gap between the performance of each model on the SMM4H evaluation set, and (2) there was a significant decrease in performance on both evaluation sets compared to the validation set. More detailed discussion of these observations follows.

First, while the gap in F1 scores on the WEB-RADR evaluation set seems similar to the gap with the validation set, there was a significant gap between the F1 score of the 2 models on the SMM4H evaluation set. CLAPA’s F1 score was 0.44, while baCLAPA achieved an F1 score of 0.51. We believe this is because CLAPA utilizes a training set to enhance medical concept representation. That is, the model heavily relies on the training set, which may result in overfitting. BERT might help diminish this problem because of its generalizability as a language model, that is, it computes word embeddings based on the full context of a sentence given a large text corpus. Thus, incorporating BERT would help CLAPA not just to learn the context better but also not overfit the model on the training set. We plan to investigate this observation further once gold labels are released by the data set developers, or if we observe a similar result in other data sets.

Second, the performance of both CLAPA and baCLAPA was significantly lower on the evaluation sets than on the validation set. This may be partly explained by the number of tweets in which none of the drugs from the drug list were found. In addition to the total number of tweets for each data set, Table 1 also shows the number of unique drugs found using our drug list, and the number of tweets that did not have any drug names from the list. Our drug list contained 4888 drug names, including generic names and brand names. Since the collection was initially built through MedlinePlus and expanded through the training set, it covers almost all tweets, with the exception of 31 tweets that contained very specific typos, such as “vioxe” or “viox” instead of “vioxx” (the correct spelling), and were excluded from the data set. In the training set, a total of 1020 unique drugs were identified from our list. However, the number of unique drugs was lower in the evaluation sets: 688 in the SMM4H evaluation set and 685 in the WEB-RADR evaluation set. The number of drugs found in a new data set is expected to be relatively low, because the list is incomplete: our list did not cover all drug names or common typos. However, the number of tweets that did not contain any drug words was a significant portion of the WEB-RADR evaluation set. The 25,646 tweets affected by this in the WEB-RADR data set would have been considered as non–drug-relevant tweets by the models, whereas the 129 tweets in the SMM4H evaluation set would have been considered as non-relevant tweets. When the models are uncertain whether or not a tweet is drug-relevant, which depends heavily on a drug list, the prediction task may suffer.

To summarize, baCLAPA achieved an F1 score of 0.51 on the SMM4H evaluation set and 0.394 on the WEB-RADR evaluation set. BaCLAPA outperformed CLAPA on both evaluation sets, which illustrates the effectiveness of the method. We observed a gap between the performance of the models on the SMM4H evaluation set and an overall decrease in evaluation performance. This trend seems to be valid for many current ADE systems, since the average evaluation score was significantly lower than the validation score in past SMM4H tasks [11,13,25]. This shows that although the suggested improvements in baCLAPA appear to perform well, they may not generalize as well on unseen data sets for the ADE classification tasks, as also observed by Gattepaille et al [14]. Further research is necessary to evaluate the generalizability of neural network–based models on the ADE classification task.

### Principal Results for the Content Analysis of ADE Tweets

Our content analysis presents the ADE themes and a comparison between the known adverse reactions and ADE mentions to answer two questions: (1) what kinds of ADE are mentioned on Twitter? and (2) of ADEs mentioned for each known drug on Twitter, how many are also known adverse reactions listed on MedlinePlus?

#### Question 1: What Kinds of ADE Are Mentioned on Twitter?

Table 4 illustrates that there were 7 primary ADE-related themes within the 941 tweets available for the qualitative content analysis. Other themes that were found but not included in the table because of their infrequency include those related to jitters, body weight, skin, sexual health, digestion, and seizure. Similarly, other ADE themes, such as those related to vision and breathing, may also be found for specific drugs.

#### Question 2: Of ADEs Mentioned for Each Known Drug on Twitter, How Many Are Also Known Adverse Reactions Listed on MedlinePlus?

Figure 2 shows the top 10 drugs and their associated ADE themes found in MedlinePlus compared to mentions on Twitter. Based on these 10 drugs, the Twitter data covered an average of 69.6% of the known adverse reactions on MedlinePlus. When we set the number of tweets to be 30 or more, the average coverage increased to 78.4%. Based on the tweet counts, we conclude that Twitter data can adequately identify known adverse reactions for most drugs. However, this depends on the number of tweets extracted for each drug. For example, when we extracted fewer than 20 tweets, the model identified less than half of the known adverse reaction themes. Setting an appropriate minimum threshold may be a critical step for such exploratory analyses.

**Figure 3:**
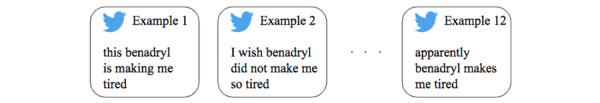
Paraphrased examples of adverse drug event themes related to Benadryl and tiredness.

Finally, social media analysis can help highlight potentially new adverse reactions from drugs. For example, Figure 3 shows tweets that pair the Benadryl and tiredness-related ADE themes, which has not been reported as a known adverse reaction in MedlinePlus, but is expressed in these tweets. Looking at specific examples of tweets can help further elaborate on these as-yet-unreported pairings. These examples could be directly updated with a reporting system such as FAERS.

In-depth analysis of social media to detect ADE mentions could also show how laypersons report ADEs in their own language. Learning such expressions could help fill a vocabulary gap between patients and health professionals and enable better communication when prescribing a drug and analyzing patient-reported outcomes. Lastly, we observe that Figure 2 presents 12 new possible pairings. These occurrences could signal the need for a potential testing of ADE hypotheses derived from in-depth social media analysis.

Through this study, we have found that Twitter covers a sufficiently wide range of ADEs given a set of drugs and also covers known adverse reactions relatively well, especially when a sufficient number of drug-related tweets are analyzed. Therefore, this study demonstrates that social media can be a reliable data source for collecting ADE mentions.

### Limitations

Our NLP system and study have some limitations. First, we did not focus on any causality relationships between a drug and an ADE. Although our qualitative analysis may signal the need for hypothesis testing, validating such claims of causality is beyond the scope of this work. Second, one of the long-term goals for this line of research is to build an automated system to collect actual ADE mentions from social media. While the classification model helps filter out large-scale data, it does not provide the actual extent of such mentions, which prevents obtaining further information, such as pairs of drug–ADE mentions, from the filtered data. To extract such mentions from tweets, we plan to work on developing an ADE extraction model. Lastly, our system cannot yet be fully deployed in practice. Our experimental results suggest that further research and development is necessary to fine-tune the models for better generalizability.

The approach presented in this paper serves as an analytical tool to identify potential adverse events in data from Twitter and other social media. It highlights both a way to validate some of the known ADEs and uncover additional potential ADEs. However, it does not fully demonstrate the relevance of social media as an independent and comprehensive source for identifying ADEs. Since there are no “gold standard” labeled data sets on possible adverse events related to a particular drug, none of the existing approaches present a comprehensive solution to the challenge of identifying all known and unknown adverse events related to a particular drug.

Further, our analysis is also biased because of the demographics of Twitter users and the differential coverage of drugs and their adverse events on Twitter. Twitter users are typically younger and more technically savvy [32]. This is especially relevant for studies of population health, since individuals from a lower socioeconomic status, underrepresented minorities, older adults, and individuals with chronic conditions are less likely to tweet [29]. Similarly, there could have been bias in the coverage of drugs and their adverse events. Although the analysis was ultimately based on 28.8 million tweets, the data were collected for the purpose of a community-based study from the Detroit metropolitan area. Tweeters in this area may discuss a particular drug more or less often than those in other communities or regions. Thus, the representation of drug usage in our data may be different from the representation of tweets collected, regardless of geographic location, making our analysis unrepresentative of overall drug usage and the types of drugs mentioned on Twitter. Rather, our analysis is limited to a certain set of drugs and their ADE mentions. However, the methodology and analysis could be repeated for other drugs.

### Conclusion

In this paper, we present a neural network–based model, baCLAPA, which incorporates a representation generated by BERT with one by CLAPA. Our experimental results demonstrate that baCLAPA outperformed CLAPA. The weak performance on unseen data signals that there is still room for improvement for the ADE classification task. Our validation study suggests that Twitter data not only include a sufficiently wide range of ADE mentions but also cover most known adverse reactions for drugs found in the relevant tweets.

Even though our work does not show any causal relationships between the drugs and ADEs mentioned, it provides possible directions to advance ADE-related work. For example, our qualitative analysis of ADE tweets could provide a basis for potential analyses and applications. It also implies that social media data can provide meaningful measurements once we have an all-purpose NLP system for collecting ADE mentions, including not just classification but also extraction. Our work demonstrates that social media can be a reliable data source for this purpose. While recent studies have developed and improved such systems, our work suggests that ADE classification systems need further research to study their robustness and reliability.

## Abbreviations

ADE: adverse drug event
baCLAPA: bidirectional encoder representations from transformers–assisted collocated long short-term memory with attentive pooling and aggregated representation
BERT: bidirectional encoder representations from transformers
CLAPA: collocated long short-term memory with attentive pooling and aggregated representation
LSTM: long short-term memory
NLP: natural language processing
SMM4H: Social Media Mining For Health
SVM: support vector machine
WEB-RADR: web-recognizing adverse drug reactions
WHO: World Health Organization
